# Chromosomal Aneuploidies and Early Embryonic Developmental Arrest 

**DOI:** 10.22074/ijfs.2015.4550

**Published:** 2015-10-31

**Authors:** Maria Maurer, Thomas Ebner, Manuela Puchner, Richard Bernhard Mayer, Omar Shebl, Peter Oppelt, Hans-Christoph Duba

**Affiliations:** 1 Landes-Frauen-und Kinderklinik Linz, Department of Human Genetics, Krankenhausstraße, Linz, Austria; 2 Johannes Kepler University, Faculty of Medicine, Linz, Austria; 3 Landes-Frauen-und Kinderklinik Linz, Department of Gynecological Endocrinology and Kinderwunsch Zentrum Linz, Krankenhausstraße, Linz, Austria

**Keywords:** Fluorescence In Situ Hybridization, Blastomeres, Embryonic Development, Aneuploidies, Chromosomes

## Abstract

**Background:**

Selecting the best embryo for transfer, with the highest chance of achieving a vital pregnancy, is a major goal in current *in vitro* fertilization (IVF) technology. The high rate of embryonic developmental arrest during IVF treatment is one of the limitations in achieving this goal. Chromosomal abnormalities are possibly linked with chromosomal arrest and selection against abnormal fertilization products. The objective of this study was to evaluate the frequency and type of chromosomal abnormalities in preimplantation embryos with developmental arrest.

**Materials and Methods:**

This cohort study included blastomeres of embryos with early developmental arrest that were biopsied and analyzed by fluorescence in-situ hybridization (FISH) with probes for chromosomes 13, 16, 18, 21 and 22. Forty-five couples undergoing IVF treatment were included, and 119 arrested embryos were biopsied. All probes were obtained from the Kinderwunsch Zentrum, Linz, Austria, between August 2009 and August 2011.

**Results:**

Of these embryos, 31.6% were normal for all chromosomes tested, and 68.4% were abnormal. Eleven embryos were uniformly aneuploid, 20 were polyploid, 3 were haploid, 11 displayed mosaicism and 22 embryos exhibited chaotic chromosomal complement.

**Conclusion:**

Nearly 70% of arrested embryos exhibit chromosomal errors, making chromosomal abnormalities a major cause of embryonic arrest and may be a further explanation for the high developmental failure rates during culture of the embryos in the IVF setting.

## Introduction

The earliest stages of human development are highly prone to error because of chromosomal abnormalities which may occur during the critical steps of meiosis, fertilization and the early cleavage stage (1,2). In vitro fertilization (IVF) allows the study of early embryonic development. Even with significantly improved culture conditions, approximately 10 to 15% of IVF embryos exhibit a permanent cell cycle arrest state, and 40% of IVF patients show at least one arrested embryo per cycle (3). Cleavage stage embryos generally show high levels of chromosomal aneuploidies, and statistically, only 1 of 5 has the capacity to implant (4). Humans are not very fertile compared to other species (5,6). Mantzouratou and Delhanty (4) postulate that on the third day of development, 60% of all IVF embryos show at least one aneuploid cell. Even embryos from patients younger than 35 years displaying the best morphology and development show an aneuploidy rate of 56% (7). Human embryos have higher aneuploidy rates than those observed during prenatal analysis and postnatal life; therefore, there appears to be strong selection pressure against chromosomally abnormal embryos. This negative selection seems to occur primarily during the pre-implantation period, most likely through developmental arrest or the degeneration of chromosomally abnormal embryos (8). 

This study specifically analyzed embryos that were not suitable for transfer. In these embryos, the aneuploidy rates are expected to be higher than in normal IVF embryos. Many studies have analyzed the correlation between morphological and developmental abnormalities in IVF embryos. A number of abnormalities are notably well correlated, such as giant oocytes (>220 µm), which are nearly always diploid, whereas other abnormalities show no direct correlation (9). 

Arrested and slow cleaving embryos are hypothesized to have higher than average rates of chromosomal abnormalities. Embryos with accelerated cleavage are also associated with a higher rate of chromosomal abnormalities, and the time frame is an important factor (2). 

Fluorescence in situ hybridization (FISH) was the method of choice used in this study. Using FISH, it was possible to distinguish between polyploidy and aneuploidy. When a representative number of blastomeres from an embryo could be analyzed, it was possible to diagnose mosaicism (9,10). 

The objective of this study was to determine whether chromosomal abnormalities are a common cause of embryonic arrest, which may be an additional explanation for the notably high developmental failure rates of IVF embryos during culture, even after continuous improvements of the culture conditions. 

## Materials and Methods

This cohort study included hundred nineteen embryos with early developmental arrest that were obtained during 45 cycles of IVF treatment. The number of embryos analyzed per cycle ranged from 1 to 9, with an average of 3 arrested embryos per cycle. All probes were obtained from the Kinderwunsch Zentrum Linz, Austria, between August 2009 and August 2011, and the genetic analyses were performed at the Department of Human Genetics, Landes-Frauen-und Kinderklinik Linz, Austria. All the arrested embryos used in this study were donated by patients undergoing conventional IVF treatment for infertility, and written consent was obtained from each patient. The study was approved by the local Ethical Review Board and was performed in accordance with the Austrian legal regulations, because only fertilization products without development potential, which would usually be discarded during normal IVF cycles, were used for analysis. The age of the patients ranged from 26 to 47 [mean age: 35 years, standard deviation (SD): 4.9 years]. Embryos were considered arrested when no cleavage had occurred during a 24-hour period (<5 cells on day 3 post fertilization, <8 cells on day 4 and <12 cells on day 5) (6,11). A percentage of all arrested embryos (47%) showed additional morphological abnormalities, such as multinucleation and uneven blastomere size. 

For the biopsy procedure, a infrared diode laser "Fertilaser" 1.48 µm (MTG, Bruckberg, Germany) was used. A hole was drilled into the zona pellucida (12,13) and the blastomeres were then separately aspirated and transferred onto glass slides. Embryos were biopsied on days 3, 4, 5 or 6, and the time of arrest ranged from day 1 to 4. Fixation was performed with ice-cold Carnoy’s fixative (3:1 methanol: acetic acid). 

Depending on the number of blastomeres per glass slide, 10-15 µl fixative was applied twice or until the cytoplasm dissolved, and the glass slide was air-dried for at least 15 minutes before the FISH procedure. 

**Fig.1:**
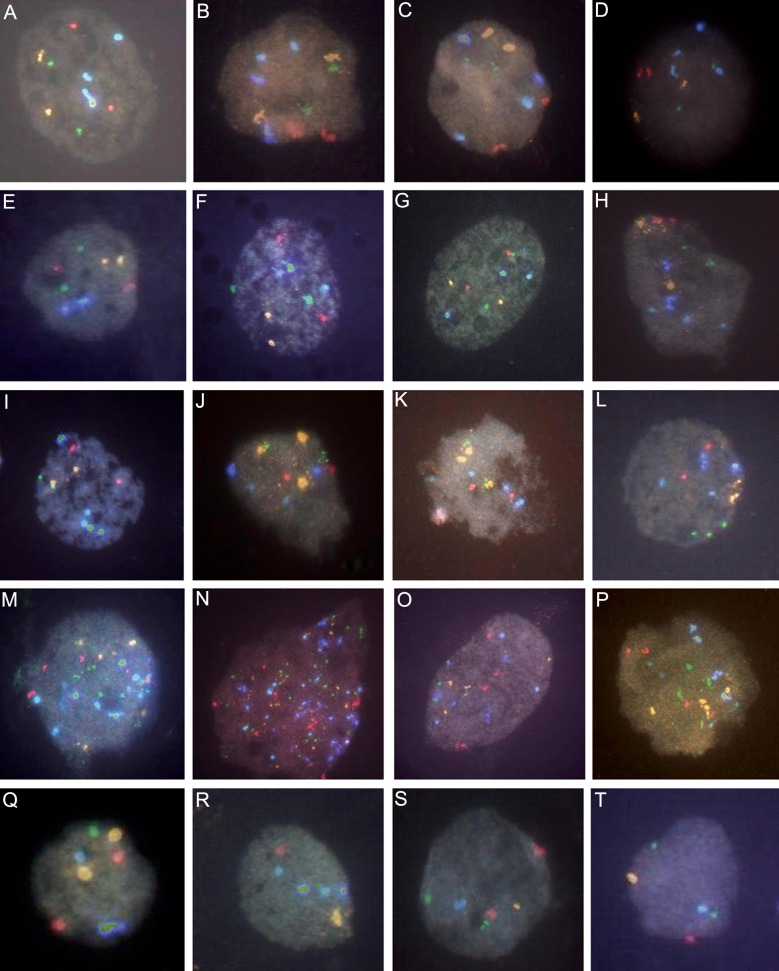
FISH images of different blastomeres displaying various abnormalities. **A-C.** Euploid blastomeres with normal FISH signal patterns for chromosomes 13 (red), 16 (aqua), 18 (blue), 21 (green) and 22 (gold), *D.* Interphase cell of a control lymphocyte, **E.** Nullisomy 16 no signal for chromosome 16 (aqua), *F-G.* Monosomy 16-only one signal for chromosome 16 (aqua), **H.** Trisomy 16-three signals for chromosome 16 (aqua), **I.** Trisomy 18-three signals for chromosome 18 (blue), **J-K.** Trisomy 22-three signals for chromosome 22 (gold), **L.** Trisomy 21-three signals for chromosome 21 (green), **M-P.** Different types of polyploidy and **Q-T.** (chaotic) mosaic (haploid/diploid).

The FISH process was performed in one hybridization round with the MultiVysion DNA Probe Panel (Vysis, Abbott Molecular Inc., Des Plaines, USA) for chromosomes 13 (LSI 13, SpectrumRed, 13q14), 16 (CEP 16 satellite II, SpectrumAqua, 16q11.2), 18 (CEP 18 alpha satellite, SpectrumBlue, 18p11.1-q11.1), 21 (LSI 21, SpectrumGreen, 21q11.2 – q22.2) and 22 (LSI 22, SpectrumGold, 22q11.2). The probes were denatured at a melting temperature of 69˚C for 8 minutes and hybridization was performed at 37˚C overnight. Next the hybridization coverslips were removed and the slides were washed for 7 minutes at 72˚C in 0.7×saline sodium citrate (SSC)/0.3% Nonidet^®^ P-40 (NP-40: Abbott Molecular Inc., Des Plaines, USA, SSC, Invitrogen, life technologies, LifeTech Vienna, Austria) followed by a 1 minute incubation at room temperature in 2×SSC/0.1% NP-40. The slides were mounted with Antifade Solution without DAPI (Vector Laboratories, CA, USA) and fluorescence microscopy was performed using an Axioplan 2 microscope equipped with specific filters for each fluorochrome. In some cases, the split signals were problematic and usually correlated with bad blastomere morphology. The probe panel was tested on lymphocyte slides prepared by standard cytogenetic procedure, scoring for 25 metaphases and 100 interphases. The probe efficiency was 97% (Fig .1D). 

The embryos were categorized into the following 6 subgroups:

I Normal diploid (euploid) embryos with all cells showing two signals for the analyzed chromosomes.
II Homogeneously abnormal (aneuploid) embryos with either monosomy (same chromosome missing in all cells) or trisomy (three chromosomes of the same type in all cells).
III Mosaicism with embryos containing two cell lines, each representing >20% of the cells.
IV Embryos with more than two abnormalities affecting multiple chromosomes and varying from cell to cell (uncontrolled division) were categorized as chaotic (complex) (14).
V Polyploid embryos with three or more signals for each analyzed chromosome in all cells.
VI Haploid embryos with only one signal for each analyzed chromosome in all cells (15).

## Results

Forty-five couples donated their arrested embryos for this study. A total of 649 blastomeres were biopsied from 119 arrested embryos. The percentage of arrested embryos per cycle ranged from 7 to 51.4%. During the biopsy procedure 422 nucleated (65%) and 227 anucleated blastomeres were counted.

An average of 5.5 blastomeres per embryo was biopsied. Only 384 cells (59.2%) showed to be clearly analyzable because the remainder were either damaged or lost during the biopsy and fixation procedure, covered with too much cytoplasm and not analyzable, showed no detectable signal or unclear signals (spots to close to the vicinity, lysed signals, overlapping signals/ cells, or probe inefficiency), and could not be located on the glass slides.

Table 1 presents the observed chromosomal abnormalities according to the maternal age. Thirty-two percent of all analyzable embryos showed a normal (euploid) result and 68% were abnormal. Twenty-one embryos displayed inconclusive results.

A total of 67 embryos showed abnormal results, 11 (16.4%) were aneuploid for all analyzed blastomeres (5 monosomies, 4 trisomies and 2 double trisomies), 11 showed mosaicism (3 with trisomy/euploid mosaicism, 1 with polyploid/euploid mosaicism, 2 with chaotic/ euploid mosaicism, 3 with monosomy/euploid mosaicism, and 2 with trisomy/monosomy mosaicism), 3 were haploid, 20 (29.9%) were polyploid (ranging from 3 N to 25 N) and 22 (32.8%) embryos showed a chaotic chromosomal complement.

There was no preferential malsegregation of one of the analyzed chromosomes, and no statistically significant difference in the frequency or type of abnormality was observed.

Figure 1 shows the FISH images of different blastomeres displaying various types of chromosomal abnormalities.

**Table 1:** Chromosomal abnormalities according to maternal age


Age (Y)	Cycles	Number of embryos	Mean value number of arrested Embryos per cycle	Standard deviation	Number of embryos diagnosed	Inconclusive embryos	%	Normal embryos	% (of all embryos with result)	Abnormal embryos	% (of all embryos with result)	Aneuploid	% (of all abnromal embryos*)	Chaotic	% (of all abnromal embryos*)	Mosaic	% (of all abnromal embryos*)	Polyploid	% (of all abnromal embryos*)	Haploid	% (of all abnromal embryos*)

26 -30	11	39	3.5	1.9	33	6	15.4	13	39.4	20	60.6	4	20	4	20	5	25	6	30	1	5
31 -35	11	35	3.2	2.7	28	7	20	11	39.3	17	60.7	1	5.9	9	52.9	3	17.6	2	11.8	2	11.8
36 -40	15	29	1.9	1	27	2	6.9	5	18.5	22	81.5	5	22.7	7	31.8	3	13.6	7	31.8	0	0
41 -47	8	16	2	1.1	10	6	37.5	2	20	8	80	1	12.5	2	25	0	0	5	62.5	0	0
Total count∑	45	119			98	21	17.65	31	31.6	67	68.4	11	16.4	22	32.8	11	16.4	20	29.9	3	4.5


## Discussion

The high rate of chromosomal abnormalities may either be an artefact of *in vitro* manipulation induced by artificial conditions in the IVF laboratory or a physiological state of embryonic development (16).

Munné et al. (17) stated that there may be a relationship between early chromosomal disorders and specific reproductive technologies, and found different rates of mosaicism at different IVF centres ranging from 11 to 52%.

IVF methods like ovarian stimulation may influence the aneuploidy rate. Verpoest et al. (18) conducted a small study analyzing unstimulated IVF cycles with patients with a low mean age (31.4 years), but even in this cohort, the aneuploidy rate was rather high (36.4%).

Labarta et al. (19) conducted a very elegant study with oocyte donors comparing chromosomal abnormalities in unstimulated and stimulated cycles in the same patient. Intrasubject comparison revealed abnormality rates of 34.8% in unstimulated and 38.2% in stimulated cycles, leading to the conclusion that moderate ovarian stimulation in young normo-ovulatory women does not significantly increase aneuploidy rates in embryos.

The overall fertility rate of the human population is low, the natural abortion rate is very high, and even during natural conception cycles, a number of chromosomal abnormalities occur. Aneuploidy occurs in approximately 0.3% of all newborns and 4% of stillbirths, while 35% of spontaneous abortions exhibit chromosomal errors, leading to the estimation that 5% of all human conceptions are aneuploid (20). The range of chromosomal abnormalities in human preimplantation embryos varies from 15% to over 85% (21, 22).

Aneuploidy may occur for a number of reasons, such as the inappropriate attachment of chromosomes to the mitotic spindle, partial inactivation of spindle checkpoint proteins or the amplification of centrosomes (23).

Vanneste et al. (24) showed in a study of normal fertile couples with a risk for inherited genetic diseases that only 9% of all generated IVF embryos had a normal chromosomal complement in all blastomeres and that nearly half of the embryos had no normal blastomeres. The study of normally conceived *in vivo* embryos is not possible; therefore, artificially produced chromosomal abnormalities cannot be excluded and the *in vivo* and *in vitro* data cannot be compared. Moreover, mouse models are not very representative of humans because the aneuploidy rates of mice are low compared to humans (16).

In our cohort study, an abnormality rate of 68% was observed, and the percentage of abnormal embryos may actually be higher because only 5 chromosomes were analyzed and the apparently euploid embryos may be aneuploid for the other chromosomes not analyzed (25). 

Of all the abnormal embryos, 29.9% were polyploid [more than two haploid (n) sets of chromosomes], ranging from 3N (triploid) to 25N, and there were many chaotic mosaic polyploidies. Reasons for this observation, such as polyspermic fertilization, are unlikely because only 2 PN were observed. Triand tetraploidy were the most commonly observed ploidies. Polyploidies may be a physiological phenomenon during preimplantation development (26). Tetraploidy can arise via different mechanisms, including cell fusion, endoreduplication and cytokinesis failure (23). FISH artefacts are unlikely because the polyploid chromosome patterns involve multiple chromosomes (25). A possible explanation is that these embryos stopped cell division, but continued DNA synthesis (9,27). 

Of the analyzed arrested embryos with abnormal results, 16.4% displayed two-cell line mosaicism and 32.8% displayed chaotic mosaicism. Human embryos display a high rate of mosaicism during all developmental stages (28) and this is of great importance for preimplantation genetic diagnosis, because the chromosomal status of an embryo is determined by only a single cell from the specific embryo (29). Meiotic errors lead to complete aneuploidies, whereas mitotic malsegregation results in mosaic aneuploidies, and postzygotic mitotic errors lead to aneuploid mosaics (25). The results of this study confirm previously published results demonstrating that post-meiotic abnormalities, such as polyploidies and mosaicism increase with decreasing embryonic development and that postmeiotic abnormalities and not aneuploidies are the most frequent outcome (7). A high percentage of mosaic embryos have diploid cells as the primary cell line (29). In their study, Daphnis et al. (28) describe diploid/aneuploid mosaicism as the predominant type of mosaicism. Chaotic mosaicism is more frequent in developmentally arrested embryos, and arrested embryos show higher proportions of abnormal cells in mosaics than non-arrested embryos. The developmental potential of mosaic embryos depends on the type and proportion of non-diploid cells with the higher the number of blastomeres containing abnormalities, the smaller the developmental capacity. Diploid-haploid mosaicism in fetal tissues has never been described, leading to the conclusion that these types of embryos are eliminated during the earliest stages of embryonic development (21). 

Aneuploid mosaicism did not increase with maternal age, and the rate of mosaic embryos decreased by age instead. However, mitotic nondisjunction has been associated with maternal age (30), leading to mosaics with trisomic and monosomic cell lines, caused by a reciprocal gain or loss in daughter cells (28). Two of this type of mosaics were observed in our cohort study, both in the 35-40 year age group. 

The high rate of chaotic embryos is noteworthy, because these embryos show various chromosomal imbalances that vary from cell to cell with no clear mechanisms for the malsegregation. Furthermore, the extensive imbalances are incompatible with normal preimplantation development (21). Chaotic embryos have very low developmental competence, and development beyond implantation and to the blastocyst stage is unlikely (14); therefore, the high rate of arrested embryos is not unexpected. The high rate of aberrations and the chaotic pattern somewhat resembles the chaotic situation of cancer cell lines and should normally be avoided by cell cycle checkpoints, which would ensure normal chromosomal numbers in daughter cells. 

According to these results, evaluating only on a single cell of an eight-cell embryo is a poor representation of the entire embryo. Based on this one cell, embryos can only be graded as "normal" or "abnormal". It is not possible to distinguish between aneuploidy or mosaic embryos (7) because one cell cannot represent mosaicism (31). 

Furthermore, the day of transfer plays an important role during the selection of the most suitable embryo. When transferring on day 2, it is not possible to distinguish between slowly developing and arrested embryos (32). 

Euploid embryos have higher blastocysts rates than chromosomally abnormal embryos (1); however, extended culture to the blastocyst stage is not reliable for selecting against chromosomally abnormal embryos, because even if there is strong selection against abnormal embryos, aneuploid embryos may also survive and develop into normal blastocysts (33,34). 

## Conclusion

Arrested embryos are a good representation of the natural negative selection against aneuploid embryos prior to preimplantation; however, nearly 32% of euploid embryos in the arrested cohort demonstrate that embryonic development depends on many factors and that embryonic arrest is caused by a wide variety of factors (3), with genetics being only one of many. 

In the context of preimplantation genetic diagnosis, our study vividly demonstrates how genetically heterogeneous human embryos can be, arrested or not, and that FISH analyses of single blastomeres have significant limitations. Array-based genotyping methods (35) on other embryonic tissues may be able to overcome these problems and identify the genetic composition of preimplantation embryos and its impact on embryonic development. 
